# Accumulation of Secondary Metabolites of *Rhodiola semenovii* Boriss. In Situ in the Dynamics of Growth and Development

**DOI:** 10.3390/metabo12070622

**Published:** 2022-07-06

**Authors:** Nina V. Terletskaya, Nazym K. Korbozova, Alexander E. Grazhdannikov, Gulnaz A. Seitimova, Nataliya D. Meduntseva, Nataliya O. Kudrina

**Affiliations:** 1 Faculty of Biology and Biotechnology and Faculty of Chemistry and Chemical Technology, Al-Farabi Kazakh National University, Al-Farabi Avenue 71, 050040 Almaty, Kazakhstan; naz-ik@mail.ru (N.K.K.); sitigulnaz@mail.ru (G.A.S.); nat.mdnt@gmail.com (N.D.M.); 2 Institute of Genetic and Physiology, Al-Farabi Avenue 93, 050040 Almaty, Kazakhstan; 3 N.N. Vorozhtsov Novosibirsk Institute of Organic Chemistry, Siberian Branch of Russian Academy of Science, 630090 Novosibirsk, Russia; agrash@nioch.nsc.ru

**Keywords:** *Rhodiola semenovii*, secondary metabolites, ontogenesis, source-sink interaction

## Abstract

*Rhodiola semenovii* Boriss. (Regel and Herder) might be a promising replacement for the well-known but endangered *Rhodiola rosea* L. In this research, the metabolic profile of *R. semenovii*, including drug-active and stress-resistant components, was studied in the context of source–sink interactions in situ in the dynamics of growth and development. Gas chromatography with mass spectrometric detection and liquid chromatography methods were used. The data obtained allow for assumptions to be made about which secondary metabolites determine the level of stress resistance in *R. semenovii* at different stages of ontogeny in situ. For the first time, an expansion in the content of salidroside in the above-ground organs, with its maximum value during the period of seed maturation, and a significant decrease in its content in the root were revealed in the dynamics of vegetation. These results allow us to recommend collecting the ground component of *R. semenovii* for pharmaceutical purposes throughout the seed development stage without damaging the root system.

## 1. Introduction

*Rhodiola semenovii* Boriss. (Regel and Herder) plants have long been used in Central Asian traditional medicine as a source of adaptogenic, choleretic, tonic, anti-inflammatory, hypoglycemic, and antioxidant qualities [1,2,3,4]. *R. semenovii* has the potential to replace the well-known *Rhodiola rosea* L., which is now listed in the *Red Book* due to its medicinal properties. However, the metabolic profile of *R. semenovii* and its prospective alterations, including drug-active and stress-resistant components, has yet to be depicted in detail, including focusing on the dynamics of vegetation at various phases of growth.

It was established that the synthesis of secondary metabolites, including those valuable for pharmacy, significantly varies by plant organs depending on the stage of ontogeny [5]. In the context of physiological, biological, and functional regulation, plant metabolites are the biochemical “levers” by which the plant organism solves numerous issues throughout its life [6]. In addition, the synthesis of metabolites in a plant is a system mediated both by external influences (temperature, precipitation, solar insolation, and other environmental factors) and internal factors (stages of plant ontogeny) [7,8]. The chemotypes are more variable than phylogeny due to the presence of a delicate balance between sink and source during plant development [9,10,11]. An adjustment in chemical profiles suggests that various biosynthetic pathways are activated during development [12]. Secondary metabolite synthesis involves some ecological effects, such as a redistribution of the plant’s internal resources between growth and defense. The theory of optimal defense assumes that distinct tissues of a plant during its growth are protected from several abiotic and biotic stressors based on their value to the plant at this stage of development [13]. During ontogeny, stressors and their intensity vary, and the concentrations of secondary metabolites (SM) depending on the availability of carbon resources also vary. The very structure of a vegetative plant is a consequence of the gradual complication of the source–sink interaction [14,15,16,17,18]. 

The carbohydrates are the primary substrates used in the processes of photosynthesis and respiration, supplying the plant with energy. They likewise provide a carbon skeleton for the synthesis of protective compounds, including SM, such as flavonoids, stilbenes, lignins, etc. It is possible that carbohydrate complexes regulate the response to light, the concentration of soil nutrients, and stress, whereas internal development programs are controlled by plant hormones [19,20]. The hypothesis “general control” states that the distribution of carbon to diverse sink organs is controlled both by the demand for “sinks” (their activity and size) and by the control of “sources” of photosynthates production [21]. The assimilation of carbon in the plant, in coordination with the absorption of nitrogen, ensures the overall production of biomass, as well as the construction of carbon skeletons of biomolecules and amino acid balance with the corresponding transport processes from source to sink. The dynamic regulation of metabolism ensures an optimal targeted delivery of SM—the right “building blocks”—to emerging and developing organs and tissues [22]. A vast arrangement of plant metabolite transporters provides flexibility and adaptability in coordinating between pathways and source–sink interactions [23,24]. Plant secondary metabolites and their degradation products can also work as plant growth modulators and integrated components of metabolic networks that are dynamically formed during ontogenesis under the pressure of many environmental factors [25,26,27,28].

The integration of phenotyping and metabolic studies in the context of the source–sink interaction is emerging as a promising technique for studying genotype–environment interactions in order to identify critical factors for increasing productivity and the optimal timing of medicinal plant material collection when the content of bioactive compounds is at its highest level [29,30]. Nevertheless, despite the fact that researchers have made significant efforts to investigate the “source–sink” connection, we are still far from a thorough understanding of the source–sink interaction in plants, as well as the rational application of “source–sink interaction” results [31].

As a result, the goal of this work was to investigate the synthesis of secondary metabolites, specifically p-hydroxyphenylethyl-O—D-glucopyranoside (salidroside), which is valuable from a therapeutic standpoint and can be considered as a generic trait of *Rhodiola* species [32] in the dynamics of vegetation in situ in the foothills of the Trans-Ili Alatau (Northern Tien Shan, Kazakhstan). We expect that studying the accumulation of valuable medicinal SMs in *R. semenovii* in the context of source–acceptor interactions will reveal patterns of stress tolerance formation in this species at different stages of in situ ontogeny, as well as the optimal time for collecting *R. semenovii* as a medicinal raw material.

## 2. Results

### 2.1. Determination of Organic Compounds in Root and Shoot of R. semenovii during Vegetation 

The comparative quantitative analysis based on GC-MS spectra from the Wiley 7th edition and NIST’02 libraries made it possible to identify 35 metabolites from different classes as significantly different components in immature plant tissues of *R. semenovii*, 68 metabolites in flowering plant tissues, and 40 metabolites in adult plant tissues at *p* < 0.05 (Tables S1–S3). 

**Figure 1:**
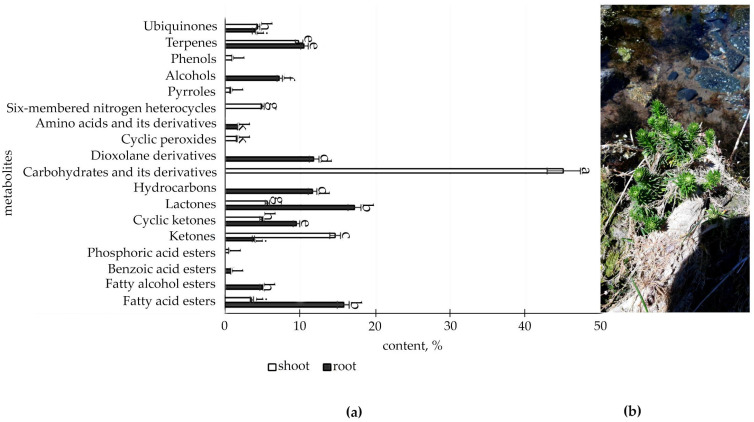
(**a**) Content of secondary metabolites classes of immature plants *R. semenovii*, %. Values presented are means (± SD). Different letters above the bars represent significant differences at *p* ≤ 0.05, *n* = 3 plants in each of 3 replicates for all treatments; (**b**) immature plants *R. semenovii*.

**Figure 2:**
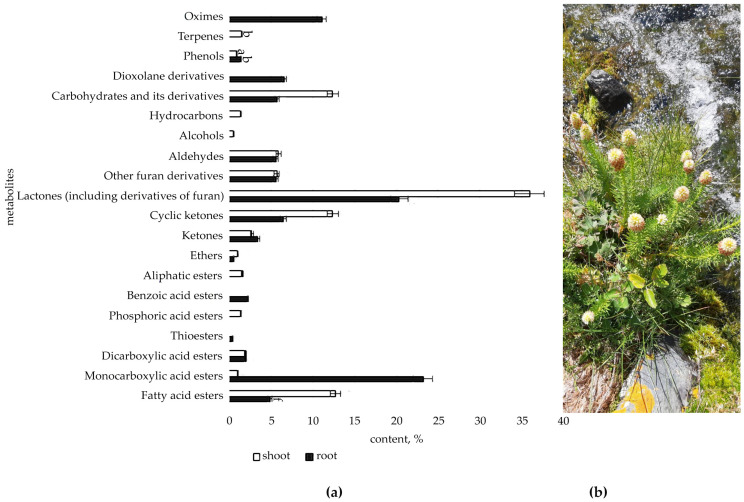
(**a**) Content of secondary metabolites classes in root and shoot of flowering plants *R. semenovii*, %. Values presented are means (±SD). Different letters above the bars represent significant differences at *p* ≤ 0.05, *n* = 3 plants in each of 3 replicates for all treatments; (**b**) flowering plants *R. semenovii*.

**Figure 3:**
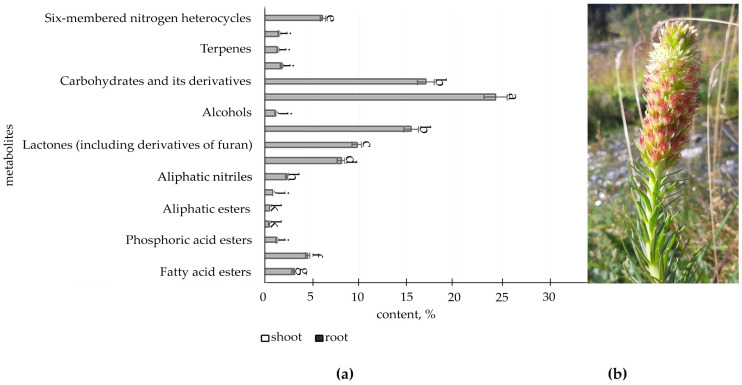
(**a**) Content of secondary metabolites classes in flowers of *R. semenovii*, %. Values presented are means (±SD). Different letters above the bars represent significant differences at *p* ≤ 0.05, *n* = 3 plants in each of 3 replicates for all treatments; (**b**) flower of *R. semenovii*.

**Figure 4:**
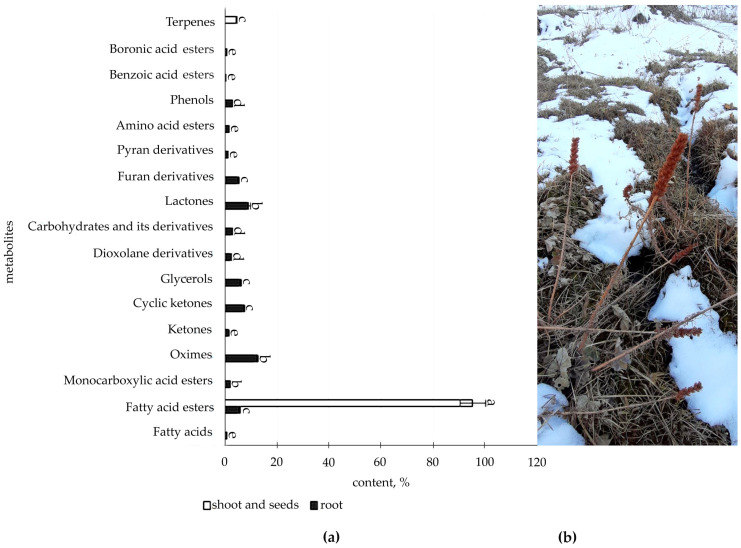
(**a**) Content of secondary metabolites classes of adult plants *R. semenovii* under ripening of seeds, %. Values presented are means (±SD). Different letters above the bars represent significant differences at *p* ≤ 0.05, *n* = 3 plants in each of 3 replicates for all treatments; (**b**) adult plants *R. semenovii* with seeds.

The metabolic profile of the root and shoot of *R. semenovii* (Figure 1, Figure 2, Figure 3 and Figure 4) under natural vegetation circumstances demonstrated quantitative variations in the classes of organic molecules at different phases of ontogenesis.

According to the data (Figure 1, Figures S1 and S2, Table S1), immature plants have a higher concentration of SM in the root, with the largest proportion found in fatty acid esters (15.9%), ketones (13.1%, including cyclic ketones), lactones (17.2%), hydrocarbons (11.6%), dioxolane derivatives (11.8%), and terpenes (10.5%). 

However, they have a comparatively low level of beneficial secondary metabolites in the shoot at this stage of development, with the exception of ketones (18.4%, including cyclic ketones), carbohydrates and their derivatives (45.1%), six-membered aromatic heterocycles (4.9%), and pyrroles (0.6%), which are only present in the shoot.

The spectrum of SMs detected in the root and shoot of plants in the flowering phase broadened (Figure 2, Figure 3 and Figures S3–S5, Table S2). At the same time, the amount of fatty acid esters (up to 4.9%), ketones and cyclic ketones (up to 10%), and dioxolanone derivatives (up to 6.6%) decreased, while hydrocarbons and terpenes were absent. The amount of lactones (including furan derivatives) increased to 20.5%. In addition, a rather substantial number of monocarboxylic acid esters (23.2%) and oximes (11.4%) were identified, which had not before been observed in the root.

It was indicated that the shoot spectrum is both quantitatively and qualitatively more diverse and affluent than the root spectrum at this stage of the growing season. The maximum in the shoot falls on lactones (including derivatives of furan) (45.6%), fatty acid esters (15.8%), ketones and cyclic ketones (15.2%), and carbohydrates and its derivatives (29.4%). At the same time, a large number of the identified metabolites in the shoot fell on flowers, such as, and in particular, hydrocarbons (24.3%), carbohydrates and its derivatives (17.0%), aldehydes (15.4%), lactones (including derivatives of furan) (9.7%), and ketones (8.1 %). The hydroxypyridines (alkaloids) (6.2%), salicylates (1.6%), and aliphatic nitriles (2.4%) were identified only in flowers.

The data illustrated in Figure 4 (Figures S6 and S7, Table S3) present organic compounds classes in the metabolic profile of adult plants *R. semenovii* under the ripening of seeds. 

According to the information provided, at this stage of development in the root, quantitative GC-MS analysis allows for the identification of a reasonably substantial content of class of components (16), with oximes (12.3%), lactones (9.3%), and ketones being the most prevalent (8.7% including cyclic ketones), whereas only fatty acids esters (95.3%) and terpenes (4.7%) were identified in the shoot and seeds.

**Figure 5:**
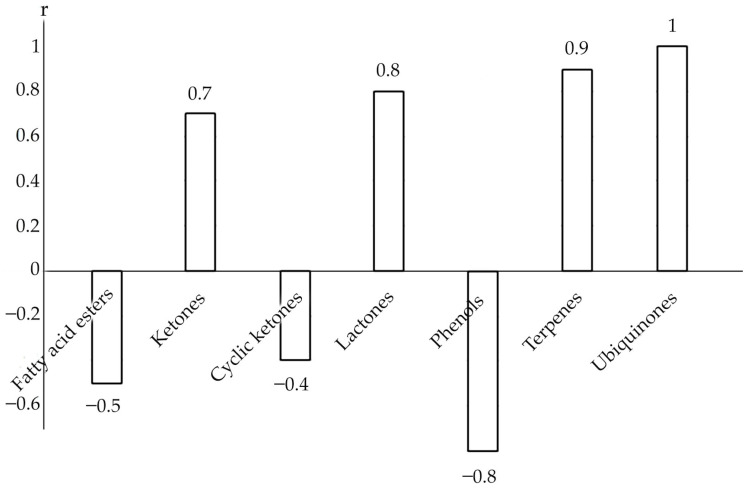
Influence of the vegetation phase on the correlation relationships of the accumulation of individual groups of SM in the root and shoot of *R. semenovii*.

Some regularities were discovered that are subject to the main groups of SM, which are present throughout the growth season in both the root and shoot, with a sufficiently high degree of correlation (Figure 5).

Thus, over the growing season, the accumulation of fatty acid esters in root declines and the proportion of their content in shoot grows (r = −0.5), but the accumulation of phenols has an inverse dependency (r = −0.8), with a maximum concentration in the root at the stage of seed maturation.

The highest concentration of ketones, terpenes, and ubiquinones was observed in the root and shoot of immature *R. semenovii* plants, with a further reduction over the growth season (correlation coefficient in the root and shoot r = 0.7, r = 0.9, and r = 1, respectively), whereas the peak of lactone accumulation in both the root and shoot of *R. semenovii* was seen during the blooming period (r = 0.8).

The remaining SM classes are absent at all phases of vegetative development in the root and shoot, with different dynamics. This implies that source–sink interactions have an unequivocal impact on the synthesis and redistribution of SM in *R. semenovii* plants during the growth season.

### 2.2. Determination of Salidrosid in Root and Shoot of R. semenovii during Vegetation

**Figure 6:**
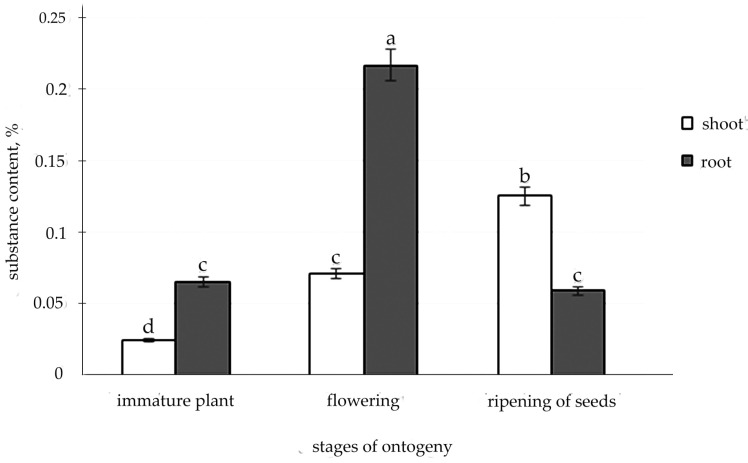
The content of salidroside in the tissues of *R. semenovii* in ontogenesis. Values presented are means (±SD). Different letters above the bars represent significant differences at *p* ≤ 0.05, *n* = 3 plants in each of 3 replicates for all treatments.

The liquid chromatography technique was used to detect the presence of the valuable medicine glycoside salidroside in the organs of *R. semenovii.* Additionally, it was found that salidroside during the growing season can be localized both in the root and shoot of *R. semenovii* in concentrations that vary depending on the stage of development (Figure 6 and Figure S8–S16).

The highest content of salidroside was noted in the root during flowering and in the shoot during seed maturation. At the same time, a positive dynamic of the accumulation of salidroside was exposed in the shoot during the growing season, whereas, after the peak level during flowering in the root, a further decrease in the concentration of this substance was indicated.

## 3. Discussion

The experiment demonstrated changes in the synthesis and redistribution of SM in *R. semenovii* plants over the growth season, indicating the impact of source–sink interactions on metabolism. In doing so, the “source” and “sink” modifications are rather conditional on the type and growth stage of the plant [31]. Therefore, the comparatively low amount of beneficial secondary metabolites, particularly in the shoots of immature plants, was clearly reflected in our experiment on the detection of SM, indicating that young plants have an increased requirement for primary metabolites and nutrients [33,34]. Meanwhile, an increased expression of the majority of detected SMs in the root may indicate other activities of the compounds, such as stimulation of the root’s capacity to absorb micronutrients [35], including in situations of insufficient water supply. This is particularly evidenced by our identification in the roots of immature plants of lipophilic alkane hydrocarbons, such as geneucosan and tetratetracontan (11.6%), which, according to the literature, are characteristic of arid regions [36].

The root is the solitary source of inorganic nitrogen, the basis of proteins and nucleic acids. Organic forms of nitrogen are frequently shaped by incorporating the ammonium ion into the composition of amino groups and amide groups through reactions already catalyzed by numerous enzymes in the early stages of plant development. Nitrogen can then pass into several carbon compounds, having been included in the composition of an organic compound. The carbon compounds accumulating in various sink organs play a significant role both as structural components of cells and as participants in respiratory metabolism [37]. This is attested by the amino acids and its derivatives identified by us in the root and shoot and six-membered nitrogen heterocycles (1.8% in the shoot) and pyrroles (0.6%) in the shoot.

We observed the highest terpene concentration in *R. semenovii* plants during the initial stages of development. The biosynthesis of isoprenoids (terpenoids) occurs in the plant under intense insolation, a high temperature, and a low concentration of carbon dioxide in the air by combining five-carbon fragments. Without its participation, the processes of plant growth and development are impossible, since many phytohormones belong to this class of compounds [37,38,39]. Thus, 3,7,11,15-tetramethyl-2-hexadecen-1-ol (9.8%), identified by us in the shoot, which can be considered as a hydrogenated diterpene alcohol, is a part of chlorophyll. We assume that the shifting of squalene synthetase in photosynthetic immature plants at the phase of squalene and supraene (10.5%) identified in the root and shoot activates a specific isoprenoid pathway for sterol synthesis in *R. semenovii*. Sterols, in turn, affect the mobility of the bilayer of cell membranes, reduce their fluidity and permeability, adjust the phase transitions of phospholipids, and influence the movement of dissolved substances and cell components through the bilayer [33]. In the meantime, the main role of terpenoids is to protect plants from various adverse environmental effects, including macro- and micro-pests [28,40,41]. However, we believe that the additional reduction in sterol synthesis during ontogenesis indicated by our study is connected with the regulation of squalene synthetase activity. This is presumably a key moment in the control of carbon flux and the formation of end products [42]. 

We identified ubiquinones, such as tocopherols, exclusively in the root and shoot of *R. semenovii* immature plants. Tocopherols in immature plants, as previously said, can serve as antioxidants in oxidative reactions under diverse abiotic conditions [4], producing phenoxyl radicals and interacting with peroxide radicals to create quinones, dimers, and trimers [43]. The same processes appear to work in natural unstable natural environments, leading to the transition of ubiquinones.

As our experimental data reveal, there is a shift in the attractor activity of organs through the ontogenesis process, which represents the stages of the execution of the morphogenetic program of development. This is clearly demonstrated by the discovered negative association between the concentration of fatty acid esters in the root and shoot of *R. semenovii*. While FAs are essential components of all plant cells [44] and precursors of the key plant hormones involved in stress tolerance [45], the significance of their highest concentration in the immature root and shoot during seed development in mature plants is undeniable.

In herbaceous perennials, such as *R. semenovii*, the transition to a new level of source–sink interaction underlies essential processes and characteristics, such as flowering, reproductive ability, and, of course, resistance to multiple stressors, since the movement of carbohydrates from “source” organs provides the substrates required for the growth of “sink” organs [46,47,48]. In this way, phosphoric acid esters were detected in immature plants *R. semenovii* in the shoot (0.39%) and in flowering plants in the shoot and in flowers (1.42 and 1.36%, respectively). In our opinion, this fact suggests that, when the primary products of photosynthesis are spent on respiration, the energy stored in the form of high-energy ester bonds of phosphoric acid is used in growth processes to maintain the status quo.

Photosynthesis and carbon and nitrogen metabolism, as well as pyruvate and glycolysis/gluconeogenesis, are considered to be the processes that have the most impact on source–sink interactions and the modifications connected with them [49]. We can assume from the experimental data that the highest levels of carbohydrates and its derivatives (12.4%) and hydrocarbons (1.4%), as well as aldehydes (5.9%) and ketones, including cyclic ketones (15.2%), are in the leaves of flowering plants *R. semenovii*, because the functioning photosynthetic leaves and other green tissues during reproductive growth are a main source of carbon and organic nitrogen. In the meantime, as we can see, the roots accumulating carbon in the form of carbohydrates and its derivatives (5.8%), mono- and dicarboxylic acid esters (25.2%), phenols (1.4%), and nitrogen in the form of oximes (11.1%) become one of the competing colors of sink photosynthates. The cells of the parenchyma and phloem of the root at this stage of ontogenesis can perform as a reserve pool for the temporary storage of C and N. If, before seed setting, they act as a sink, then, during seed setting, they frequently play the role of a source [50,51]. Thus, it was indicated in the literature that histochemical studies revealed phenolic substances in the rhizomes and roots of *R. rosea* that were localized not only in parenchymal, but in vascular tissues as well [52].

If the main function of leaves during the flowering stage is to fix CO_2_ through photosynthesis (source), then flowers (as a sink) are largely dependent on organic molecules synthesized in leaves and roots [53]. Nevertheless, flowers are not just a sink of carbohydrates and amino acids. As presented in the literature, they utilize them as a basis for the synthesis of enzymes and structural proteins, as well as precursors of nitrogen-rich secondary metabolites and signaling molecules [54,55,56]. We are aware of the possibility of increasing the level of amino acids in flowers in response to heat stress [57]. However, a detailed analysis of the metabolism of nitrogenous compounds at the flowering stage does not exist yet. According to our results, a relatively high content of hydroxypyridines (alkaloids) (6.2%) and aliphatic nitriles (2.4%) in *R. semenovii* flowers is presented. These alkaloids can be involved in a “sourcesink interaction” in a certain balance between distribution and degradation in the production of amino acids [58]. Moreover, aliphatic nitriles decompose under physiological conditions with the formation of the corresponding carbonyl compound and hydrocyanic acid, which induces the toxicity of plants and their protection from being consumed by animals.

Fundamentally, flower nectar is an aqueous solution of sucrose, glucose, and fructose [52]. Among other things, this is likewise confirmed by our experimental data demonstrating an almost 17% sucrose level in the *R. semenovii* flower. According to Southwick, nectar is an extremely powerful sink [59]. The energy used to produce flower nectar is almost double that stored in seeds. It is known that the level of complexity of communication between pollen and pistil makes the pollination process extremely susceptible to numerous stress factors [60]. However, the role of metabolism and hormonal signaling in conferring tolerance to abiotic stresses at this stage of ontogeny has not been fully studied so far [61]. We assume that the level of stress tolerance of a *R. semenovii* flower can primarily determine the presence of terpenoids (1.4%), lactones (9.7%), phenols (1.8%), and alcane such as heneicosane (21.3%) detected by this experiment, which, according to the literature, quantitatively depends on the type of habitat [61]. Furthermore, signaling molecules formed from the group of polyunsaturated fatty acids are involved in the formation of body responses to various environmental signals [4,62].

With a sufficient long-term supply of assimilates, particularly nitrogen, from the roots at the late stages of vegetation, a mild stimulating effect on developing seeds may occur. The seeds perform the function of the main sink, which activates the formation of the endosperm [63]. The enhanced sink strength in late grain filling stages can eventually lead to the higher conversion efficiency of stem and shell stocks for grain filling as well [64]. Our experiments revealed an increased content of fatty acids esters (95.3%) and terpenes (4.7%) in shoot and seeds at this stage of ontogenesis. The obtained results also confirm the opinion that, by the end of the seed formation phase (as the power of absorption of the latter increases), the supply of assimilates to the roots reduces [65]. On this metabolic spectrum, biologically active oximes (12.3%), lactones (9.3%), cyclic ketones (7.2%), alcohols (6%), and furan derivatives (5.1%) dominate in the root. We have previously detected elevated concentrations of some of them under the influence of induced cold stress [4]. Thus, we can refer to the active formation of the stored SM pool, since cold exposure in situ is a natural phenomenon at this stage of ontogeny and the active vegetation of terrestrial organs is terminated.

According to Suzich et al. [66], up to 25% of the total carbon flux in higher plants passes through the shikimate pathway. A significant part of one of the final products of this metabolic pathway is consumed in the formation of the carbon skeleton of phenols and the accumulation of phenolic compounds [5]. Meanwhile, temperature, precipitation, or solar insolation do not directly influence the content of phenolic compounds in plant tissues. However, they can influence the signaling substances that regulate the transitions of ontogeny stages, which, in turn, causes an adjustment in the biochemical composition [67]. In general, we ascertained a change in the content of phenols in the organs of *R. semenovii* in the dynamics of vegetation, with their maximum content in the root at the stage of seed maturation. At the same time, we identified them in the shoot of immature plants (0.83%); in the root, the shoot, and flowers (1.39, 0.93, and 1. 81%, respectively) of flowering plants, and in the root (3.01%) of adult plants during seed maturation.

Molecules of the valuable phenolic chemical “salidroside,” which contains a benzene ring, are also products of a specific metabolism along the shikimate pathway. According to the literature, the high content of phenolic compounds, which include salidroside, and their most dramatic quantitative changes are observed during periods of the greatest intensity of plant life functions [68,69]. It is observed in the literature that the maximum level of accumulation of such SMs as essential oils and steroidal glycosides often falls in the period of budding and flowering [68]. Kiryanov et al. [70] and Kurkin et al. [71] hence recorded the maximum accumulation of salidroside in the root of *R. rosea* in the flowering phase, which is consistent with the data of our study on the accumulation of salidroside in the root of *R. semenovii* during this period. Our results are likewise consistent with those of Kim [72] and Rybakova [59] on the presence of salidroside in the shoot of *R. rosea* during flowering, with amounts ranging from 0.08 to 0.74% based on the dry weight. 

At the same time, we discovered positive dynamics of salidroside accumulation in the shoot of *R. semenovii* during the growing season, with a maximum during seed maturation, which can serve as a basis for recommending plant material collection during this period without damaging the plant’s root system.

Consequently, the analysis of multidirectional and coordinated source–sink interactions determines the correctness of this approach toward the complex study of the integral metabolism of a plant organism in the dynamics of vegetation in situ. This approach may lead to a further comprehension of the source–sink interaction and, in turn, contribute to the more optimal timing of the collection of medicinal plant materials. In the future, this method may contribute to the rational manipulation of “source–sink” mechanisms to identify new alternative opportunities for obtaining valuable herbal medicines.

## 4. Materials and Methods

### 4.1. Plant Material

*Rhodiola semenovii* (Regel and Herder) Boriss. plants are immobile tap-rooted vegetative short-rhizome perennials. The inflorescence is a long, dense spiky raceme. Flowering occurs 3 to 4 years after sowing [73,74,75]. *R. semenovii* prefers high soil moisture and a sunlight location [76]. Experimental plants were selected at an altitude of 2350 m above sea level in the foothills of the Trans-Ili Alatau (latitude: 43°04′21″ N, longitude: 76°59′07″ E) in the dynamics of vegetation (10 May, 10 July, 5 September). The average annual air temperature at this altitude was +3.12 °C and precipitation was, on average, 881 mm per year [77].

Plant material of *R. semenovii* was identified in the Republican State Enterprise at the Institute of Botany and Phytointroduction of the Ministry of Education and Science of the Republic of Kazakhstan. Plant number of *R. semenovii* in the seed bank—3885. 

Each analyzed group of plant tissues consisted of one shoot and a part of the rhizome from each of the three plants of the same age growing close to each other to ensure the reliability of the data obtained and minimal damage to the living plants.

### 4.2. Determination of Organic Compounds in Extracts

One of the best methods for identifying SM is gas chromatography–mass spectrometry (GC-MS), which allows for the isolation and analysis of compounds in a single step using a mass detector and available GC-MS libraries [78]. Gas chromatography with mass spectrometric detection (Agilent 6890 N/5973 N, Santa Clara, CA, USA) was used for determination of organic compounds. For this experiment, plant tissue samples were fixed in 96% ethanol at a ratio of 100 g of tissue: 500 mL of ethanol. The extraction was carried out in an orbital shaker in two stages (72 h each) with the same solvent until a clear colorless solvent was obtained. Sample volume 1.0 µL, sample injection temperature 260 °C, without flow division. Each sample was injected into the GC-MS system one time (three technical repetitions in total). Separation was carried out using a chromatographic capillary column DB-35 MS with a length of 30 m, an inner diameter of 0.25 mm, and a film thickness of 0.25 µm at a constant carrier gas (helium) velocity of 1 mL/min. The chromatographic temperature was programmed from 40 (exposure 0 min) to 150 °C with a heating rate of 10 °C/min (exposure 0 min) and up to 300 °C with a heating rate of 5 °C/min (exposure 10 min). Detection was carried out in the SCAN *m*/*z* 34–850 mode. Agilent MSD ChemStation software (version 1701EA) (Santa Clara, CA, USA) was used to control the gas chromatography system and to register and process the obtained results and data. For data processing, the average values of the obtained data were taken. Data processing included determination of retention times and peak areas, as well as processing; spectral information was obtained using a mass spectrometric detector. The Wiley 7th edition and NIST’02 libraries were used to decode the obtained mass spectra (the total number of spectra in the libraries is more than 550 thousand). The chromatograms and tables are presented in the Supplementary Materials (Tables S1–S3, Figures S1–S7).

### 4.3. Determination of Salidrosid

Liquid chromatography (LC) is a highly selective, but extremely flexible and very sparing method for isolating secondary metabolites from plants with weak chromophores [79]. Water–alcohol extracts of *R. semenovii* organs were kept at a temperature of +40–50 °C. The extracts were evaporated to a constant weight of dry residues (within 10–12 h). Liquid chromatography was carried out on a Milichrom-A-02 liquid chromatograph (“EcoNova” JSC, Novosibirsk, Russian Federation) with a chromatographic column 75 × 2 mm filled with a ProntoSIL-120-5-C18 sorbent. A total of 20 μL of the dried extract solution (solvent MeOH-H_2_O, 2:1) was collected into the needle of the sample injection device; the sample was injected into the device. Chromatography was performed in gradient mode. The eluent feed rate was 200 µL/min. Detection was under-taken at a wavelength of 220, 280 nm. Gradient composition: 300 µL methanol–0.05 N H_3_PO_4_ mixture (1:9, *v*/*v*)—2200 µL methanol–0.05 N H_3_PO_4_ mixture (11:9, *v*/*v*); further, a gradual increase in the concentration of methanol to pure. The duration of the analysis was 20 min. The retention time of tyrosol was 6.5 to 7.5 min. The limit of detection of tyrosol in the described experiments was 0.005% (for the dry part of the extract). The location of the tyrosol peak on the chromatogram was determined by the addition method. The concentration of tyrosol was determined in comparison with the chromatogram of pure tyrosol (tyrosol produced by NIOCH SB RAS, SOV 98% by GLC) (Figures S8–S16). No ion chromatograms were recorded. 

The dry residue content in the extract was calculated by the formula:

1: (100 × mdry residue)/mextract 1

The tyrosol content in the dry part of extract 1 was calculated by the formula:

1: 100 × S tyrosol on chrom. of extr. × Ctyrosol in calibr. sol./

S tyrosol on chrom. of calibr. sol. × Ctyrosol in calibr. sol.

The content of salidroside in the dry part of extract 2 was calculated by the formula:

C tyrosol in the dry part of extr.1 × 2.17

### 4.4. Statistical Analyses

All experiments were conducted in three replicates (biological and technical). The processing of data and graphing was performed using Microsoft Excel (Microsoft Corp., Redmond, Washington, DC, USA). Atypical values were excluded from the data based on *t*-tests, and the standard error of the average sample was calculated. Correlations between expressed metabolites in the root and shoot in the dynamics of vegetation were analyzed using the Spearman coefficient. Differences were considered significant at *p* < 0.05.

## 5. Conclusions

In this work, the features of the accumulation of some secondary metabolites of the *R. semenovii* plant were exposed in the dynamics of growth and development in the context of source–sink interactions in situ. Assumptions were made about which secondary metabolites determine the level of resistance to stress in *R. semenovii* at different stages of ontogeny in situ. For the first time, the presence of seasonal changes in the content of glycosides was demonstrated, due to a significant decrease in the content of salidroside in the root during the reproductive development of plants. At the same time, the dynamics of the enlargement in the content of salidroside during the growing season in the aerial organs, with a maximum during seed ripening, was observed. As a result, it allows for recommendations on collecting medicinal raw materials in this phase without damaging the root system.

## Data Availability

Not applicable.

## Supplementary Materials

The following are available online at https://www.mdpi.com/article/10.3390/metabo12070622/s1, Table S1: Content of SM in immature plants of *R. semenovii*, Figure S1: Chromatogram of immature plants extract (roots of *R. semenovii*), Figure S2: Chromatogram of immature plants extract (shoots of *R. semenovii*), Table S2: Content of SM in flowering plants of *R. semenovii*, Figure S3: Chromatogram of flowering plants extract (roots of *R. semenovii*), Figure S4: Chromatogram of flowering plants extract (shoots of *R. semenovii*), Figure S5: Chromatogram of flowering plant extract (flowers of *R. semenovii*), Table S3: Content of SM in ripening of seeds of *R. semenovii*, Figure S6: Chromatogram of adult plants extract (roots of *R. semenovii*), Figure S7: Chromatogram of adult plants extract (shoots of *R. semenovii*), Figure S8: Chromatogram of tyrosol calibration solution-3 (220nm), Figure S9: Determination of salidroside content in the shoot of an immature plants of *R. semenovii*, Figure S10: Determination of salidroside content in the root of an immature plants of *R. semenovii,* Figure S11: Chromatogram of tyrosol calibration solution (280nm), Figure S12: Determination of salidroside content during the flowering in the shoot of the plants of *R. semenovii,* Figure S13: Chromatogram of tyrosol calibration solution-2 (220nm), Figure S14: Determination of salidroside content during the flowering in the root of the plants of *R. semenovii*, Figure S15: Determination of salidroside content during ripening of seeds in the shoot of the plants of *R. semenovii*, Figure S16: Determination of salidroside content during ripening of seeds in the root of the plants of *R. semenovii*.
