# Supplementary material for: Accumulation of Secondary Metabolites of Rhodiola semenovii Boriss. In Situ in the Dynamics of Growth and Development
Source: Metabolites. 2022 Jul 6;12(7):622. doi: 10.3390/metabo12070622 (PMC9323023; doi:10.3390/metabo12070622)
Supplement: Supplementary file 1 [file metabolites-12-00622-s001.zip › metabolites-1785259-supplementary.pdf]

Table S1. Content of SM in immature plants of *R. semenovii*

	in root			in shoot		
	Content, %	Retention time, min	Identification probability, %	Content, %	Retention time, min	Identification probability, %
Fatty acid esters						
9,12-Octadecadienoic acid, methyl ester	-	-	-	2.3	35.1	82
9,12,15-Octadecatrienoic acid, methyl ester	-	-	-	1.4	35.5	80
Ethyl 9,12,15-octadecatrienoate (Ethyl 9α-linolenate, linolenic acid ethyl ester)	5.3	35.5	88	-	-	-
Acetic acid n-octadecyl ester	10.6	42	71	-	-	-
sum	15.86			3.6		
Fatty alcohol esters						
1-Docosanol, acetate	4.9	42.0	77			
sum	4.9					
Ketones						
1-Phenyl-2-butanone	-	-	-	7.6	14.7	68
2-Propanone, 1-acetyloxy-	3.7	10.4	88	1.3	10.5	88
5-Hexen-2-one	-	-	-	5.7	14.8	80
sum	3.7			14.6		
Cyclic ketones						
4-Cyclopentene-1,3-dione	5.8	11.0	85	1.9	11.0	83
2-Cyclopenten-1-one, 2-hydroxy-	-	-	-	2.1	11.8	70
1,2-Cyclopentanedione, 3-methyl-	0.8	14.2	88	0.8	14.2	78
2-Cyclopenten-1-one, 2-hydroxy-3-methyl-	2.8	14.2	81	-	-	-
sum	9.4			4.8		
Lactones						
Butyrolactone	5.8	12.7	76	-	-	-
2-Hydroxy-gamma-butyrolactone	11.4	14.7	88	-	-	-
2(5H)-Furanone, 3-methyl-	-	-	-	5.5	14.0	91
sum	17.2			5.5		
Dioxolane derivatives						
1,3-Dioxol-2-one,4,5-dimethyl-	11.8	14.8	67	-	-	-
sum	11.8					
Hydrocarbons						
Heneicosane	4.9	39.0	85	-	-	-
Tetratetracontane	6.7	45.0	71	-	-	-
sum	11.6					

continuation of **Table S1.**						
Carbohydrates and its derivatives						
Lactose	-	-	-	35.4	36.2	70
Ethyl α-d-glucopyranoside-	-	-	-	9.7	27.8	77
sum				45.1		
Cyclic peroxides						
1,2,4,5-Tetroxane, 3,3,6,6-tetramethyl-	-	-	-	1.5	19.7	42
sum				1.5		
Amino acids and its derivatives						
l-Alanine, N-(2-furoyl)-, hexyl ester	1.6	20.1	60	-	-	-
sum	1.6					
Six-membered nitrogen heterocycles						
Pyrazine, 2,5-dimethyl-	-	-	-	1.1	10.7	81
Pyrimidine, 4,6-dimethyl-	-	-	-	0.8	10.8	69
2-Aminopyrimidine-1-oxide	-	-	-	3.0	20.9	65
sum				4.9		
Pyrroles						
3-Methyl-4-phenyl-1H-pyrrole	-	-	-	0.6	26.9	71
sum				0.6		
Alcohols						
Cyclopropyl carbinol	7.2	17.1	78			
sum	7.2					
Phenols						
Phenol, 2,6-dimethoxy-	-	-	-	0.8	21.7	63
sum				0.8		
Di- and triterpenes						
Squalene	3.2	45.2	86	7.5	45.2	92
Supraene	7.3	45.2	87	-	-	-
3,7,11,15-Tetramethyl-2-hexadecen-1-ol (phytol)	-	-	-	2.3	27.4	80
sum	10.5			9.8		
Ubiquinones						
γ-Tocopherol	2.1	49.9	60	1.3	49.9	71
Vitamin E	1.7	50.9	60	3.0	50.9	79
sum	3.8			4.3		
Benzoic acid esters						
Benzoic acid, heptyl ester	0.7	29.9	66	-	-	-
sum	0.7					
Phosphoric acid esters						
Phosphoric acid, diethyl nonyl ester	-	-	-	0.4	24.0	66
sum				0.4		

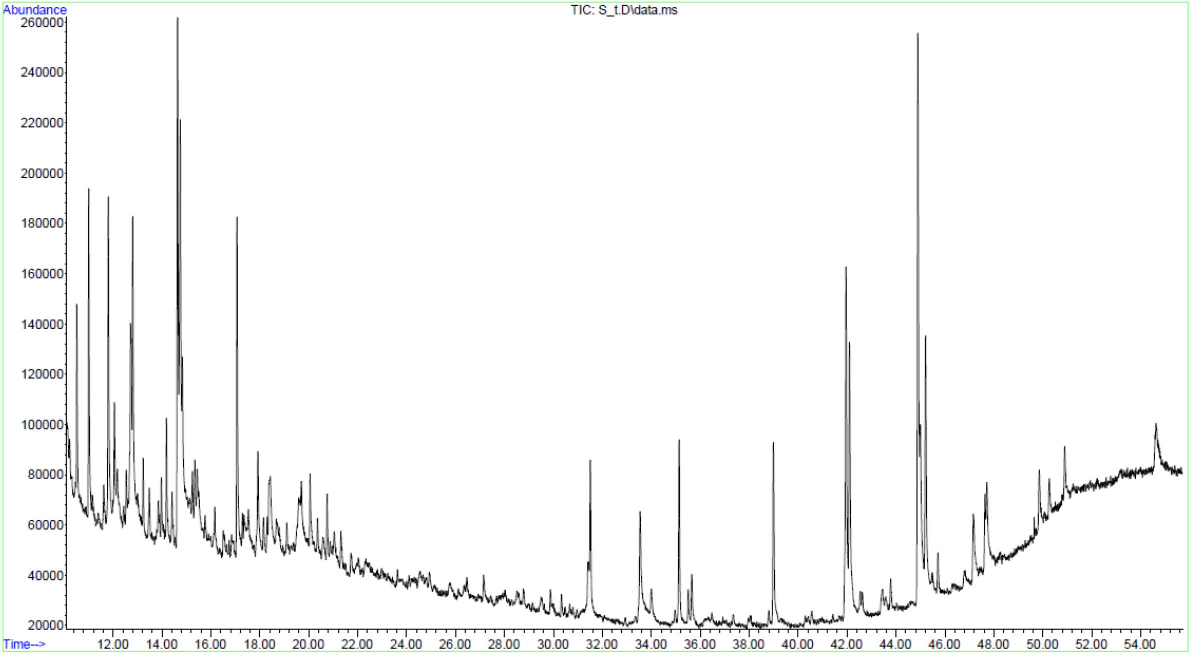

Figure S1. Chromatogram of immature plants extract (roots of *R. semenovii*)

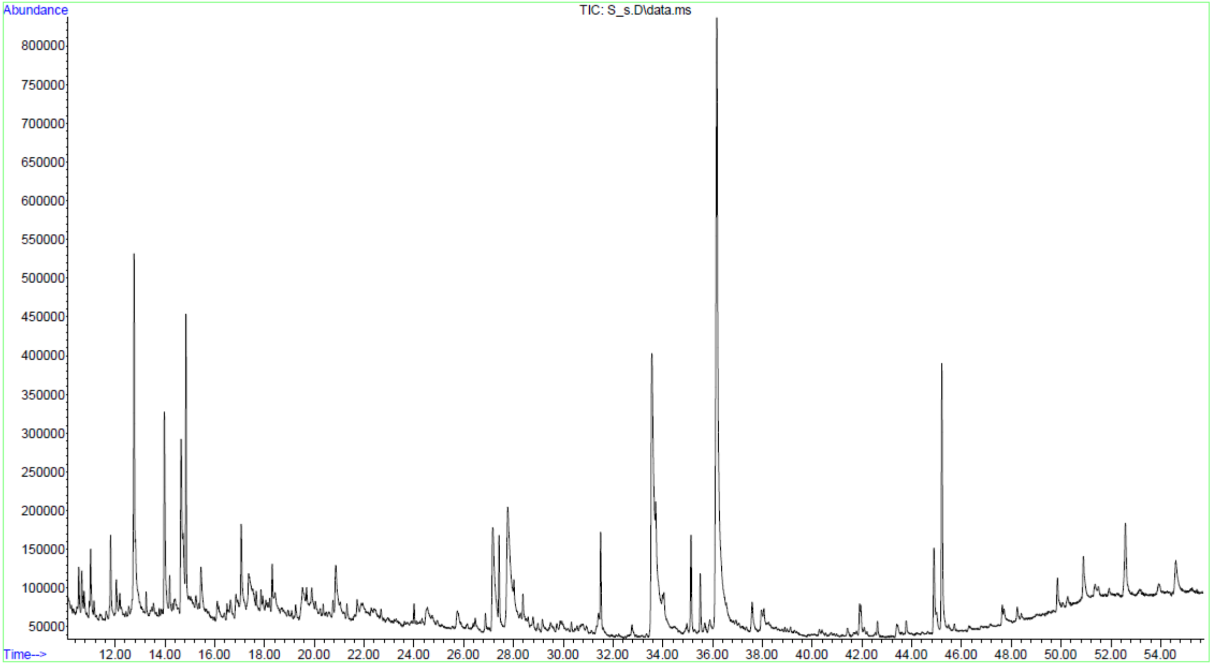

Figure S2. Chromatogram of immature plants extract (shoots of *R. semenovii*)

Table S2. Content of SM in flowering plants of *R. semenovii*

	in root			in shoot			in flovers		
	Content, %	Retention time, min	Identification probability, %	Content, %	Retention time, min	Identification probability, %	Content, %	Retention time, min	Identification probability, %
Fatty acid esters									
9,12-Octadecadienoic acid, ethyl ester	2.6	44.0	84.0	3.1	44.0	74	-	-	-
Ethyl 9,12,15-octadecatrienoate (Ethyl 9α-linolenate, linolenic acid ethyl ester)	-	-	-	5.6	44.9	87	-	-	-
Hexadecanoic acid, ethyl ester (palmitic acid ester)	2.3	38.0	89.0	4.0	38.0	88	3.1	34.0	64
sum	4.9			12.7			3.1		
Monocarboxylic acid esters									
Propanoic acid, 2-oxo-, methyl ester	22.1	10.3	89.0	-	-	-	-	-	-
Propanoic acid, propyl ester	1.1	11.6	66.0	-	-	-	-	-	-
Acetic acid, ethoxyhydroxy-, ethyl ester	-	-	-	1.05	11.6	60	1.0	11.4	61
Methoxyacetic acid, cyclobutyl ester	-	-	-	-	-	-	2.1	13.3	63
Pentanoic acid, 2-hydroxy-3-methyl-, methyl ester	-	-	-	-	-	-	1.5	13.7	61
sum	23.2			1.05			4.6		
Dicarboxylic acid esters									
3-Oxobutanoic acid methyl ester	1.5	18.6	61.0	-	-	-	-	-	-
2-(Acetyloxy)-2-methyl-3-oxobutanoic acid ethyl ester	0.5	15.6	62.0	-	-	-	-	-	-
Fumaric acid, 3-methylbut-3-enyl tridecyl ester	-	-	-	0.7	17,1	63	-	-	-
Ethanedioic acid, diethyl ester	-	-	-	1.3	15.4	85	-	-	-
sum	2.0			2.0					
Thioesters									
Propanethioic acid, S-pentyl ester	0.5	11.9	66.0	-	-	-	-	-	-
sum	0.5								
Phosphoric acid esters									
Phosphoric acid, diethyl nonyl ester	-	-	-	1.4	29.8	72	1.4	29.8	68
sum				1.4			1.4		

continuation of **Table S2.**									
Benzoic acid esters									
Benzoic acid, hept-2-yl ester	0.3	36.4	63.0	-	-	-	-	-	-
Benzoic acid, tetradecyl ester	-	-	-	-	-	-	0.5	36.7	64
Benzoic acid, pentadecyl ester	0.4	36.7	60.0	-	-	-	-	-	-
1,2-Benzenedicarboxylic acid, bis(2-methylpropyl) ester	1.6	37.9	65.0	-	-	-	-	-	-
sum	2.3						0.5		
Aliphatic esters									
Allyl acetate	-	-	-	1.6	24.3	65	-	-	-
i-Propyl 12-methyl-tridecanoate	-	-	-	-	-	-	0.6	34.3	67
sum				1.6			0.6		
Ethers									
Ethane, 1,2-bis[(4-amino-3-furazanyl)oxy]-	0.6	10.8	63.0	-	-	-	-	-	-
Pentane, 1,1-diethoxy-	-	-	-	1.0	1.,7	74	0.9	12.6	70
sum	0.6			1.0			0.9		
Aliphatic nitriles									
Propanenitrile, 3-methoxy-	-	-	-	-	-	-	2.4	13.3	60
sum							2.4		
Ketones									
2-Propanone, 1-(acetyloxy)-	2.7	12.9	82.0	-	-	-	-	-	-
5,9-Dodecadien-2-one, 6,10-dimethyl-, (E,E))-	0.8	24.3	65.0	-	-	-	-	-	-
2-Propanone, 1-acetyloxy-(2)	-	-	-	2.8	12.3	82	-	-	-
5-Hexen-2-one	-	-	-	-	-	-	5.1	17.6	68
2-Hexanone, 5-methyl-	-	-	-	-	-	-	3.0	24.3	68
sum	3.5			2.8			8.1		
Cyclic ketones									
4-Cyclopentene-1,3-dione	-	-	-	3.6	12.9	87	-	-	-
2-Cyclopenten-1-one, 2-hydroxy-	-	-	-	5.8	13.8	88	-	-	-
1,2-Cyclopentanedione	4.2	13.8	90.0	-	-	-	-	-	-
1,2-Cyclopentanedione, 3-methyl-	-	-	-	2.1	16.8	88	-	-	-
2-Cyclopenten-1-one, 2-hydroxy-3-methyl-	1.6	16.8	88.0	-	-	-	-	-	-
1,2-Cyclohexanedione	0.7	15.9	81.0	0.9	15.9	69	-	-	-
sum	6.5			12.4					

continuation of **Table S2.**									
Lactones (including derivatives of furan)									
Butyrolactone	1.7	15.0	77.0	2.3	15.0	79	1.6	14.8	63
2-Hydroxy-gamma-butyrolactone	11.9	17.5	87.0	20.9	17.5	78	-	-	-
2',3'-Dideoxyribonolactone	1.9	24.1	67.0	-	-	-	-	-	-
2(5H)-Furanone	3.9	15.1	92.0	4.3	15.1	89	1.0	14.9	67
2(5H)-Furanone, 3-methyl-	-	-	-	-	-	-	7.1	16.4	86
2,5-Dimethyl-4-hydroxy-3(2H)-furanone	-	-	-	7.5	17.6	64	-	-	-
2(3H)-Furanone, 5-acetyldihydro-	0.9	22.2	72.0	-	-	-	-	-	-
2(3H)-Furanone, 5-heptyldihydro-	-	-	-	0.9	22.2	60	-	-	-
sum	20.3			35.9			9.7		
Other furan derivatives									
2-Furanmethanol	1.8	11.2	89.0	1.9	11.2	81	-	-	-
2,5-Furandione, 3-methyl- (9)	-	-	-	-	-	-	-	-	-
Benzofuran, 2,3-dihydro-	3.8	22.45	72.0	3.8	22.5	72	-	-	-
sum	5.6			5.7					
Aldehydes									
Nonanal	-	-	-	-	-	-	2.3	17.2	74
Benzeneacetaldehyde	-	-	-	-	-	-	6.6	17.4	81
Pentanal	5.6	20.7	79	5.9	20.7	74	-	-	-
Benzaldehyde, 3-methyl-	-	-	-	-	-	-	2.5	22.5	61
Octadecanal	-	-	-	-	-	-	2.5	32.6	70
Tetradecanal	-	-	-	-	-	-	1.5	34.8	69
sum	5.6			5.9			15.4		
Alcohols									
Ethanol, 2-(9-octadecenyloxy)-, (Z)-	-	-	-	0.5	34.3	61	-	-	-
Cyclopropyl carbinol	-	-	-	-	-	-	1.2	20.6	64
sum				0.5			1.2		
Hydrocarbons									
Tetradecane	-	-	-	-	-	-	1.4	22.9	63
Heneicosane	-	-	-	-	-	-	21.3	38.7	83
Octadecane, 3-ethyl-5-(2-ethylbutyl)-	-	-	-	-	-	-	1.6	41.5	69
Heptadecane, 9-hexyl-	-	-	-	1.4	45.4	69	-	-	-
sum				1.4			24.3		
Carbohydrates and its derivatives									
Diethyl mercaptal of d-mannose	1.0	16.3	62.0	-	-	-	-	-	-
Sucrose	4.8	30.5	70.0	8.6	24.5	74	17.0	24.5	71
β-D-Glucopyranose, 1,6-anhydro-	-	-	-	3.8	31.8	87	-	-	-
sum	5.8			12.4			17.0		

continuation of **Table S2.**									
Dioxolane derivatives									
1,3-Dioxol-2-one,4,5-dimethyl-	6.6	17.7	74.0	-	-	-	-	-	-
sum	6.6								
Phenols									
Phenol	0.9	14.7	87.0	0.9	14.7	76	-	-	-
Phenol, 2,6-dimethoxy-	0.5	27.0	64.0	-	-	-	1.8	27.0	66
sum	1.4			0.9			1.8		
Di- and sesquiterpenes									
3,7,11,15-Tetramethyl-2-hexadecen-1-ol (Phytol)	-	-	-	1,51	33,7	73	0.4	33.7	68
2-Pentadecanone, 6,10,14-trimethyl-	-	-	-	-	-	-	1.0	34.9	72
sum				1.51			1.4		
Salicylates									
Homosalate	-	-	-	-	-	-	1.6	37.4	78
sum							1.6		
Oximes									
Oxime-, methoxy-phenyl-	11.1	10.9	80.0	-	-	-	-	-	-
sum	11.1								
Hydroxypyridines (alkaloids)									
2(1H)-Pyridinone, 6-hydroxy-	-	-	-	-	-	-	6.2	19.4	75
sum							6.2		

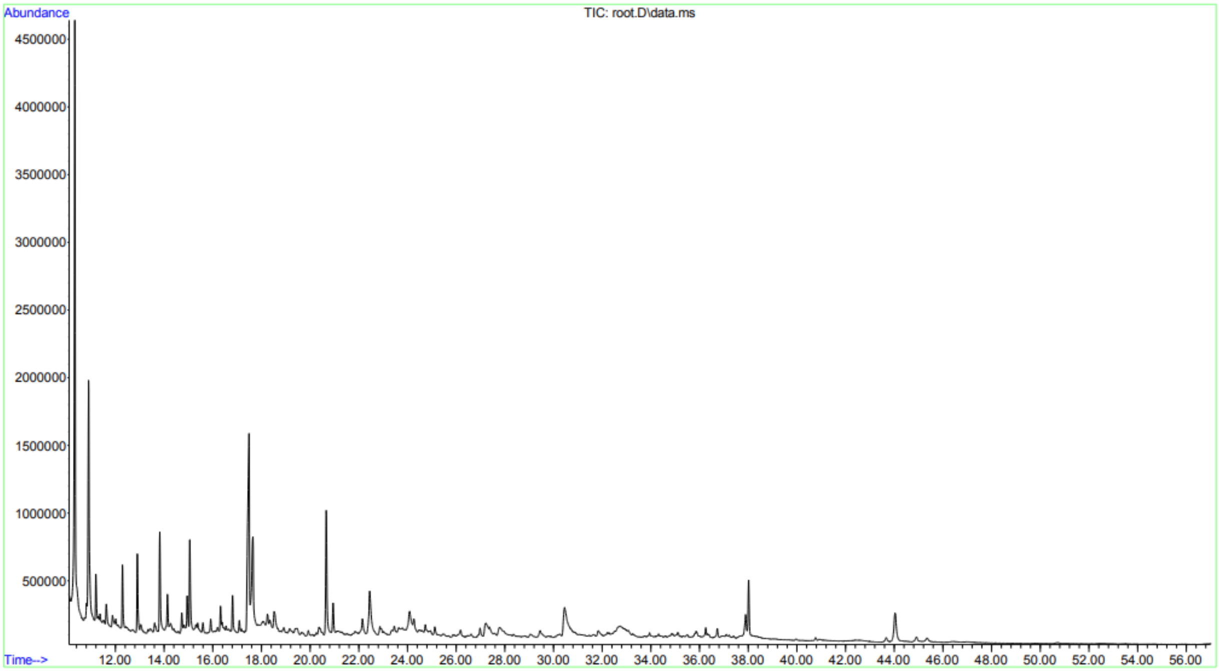

Figure S3. Chromatogram of flowering plants extract (roots of *R. semenovii*)

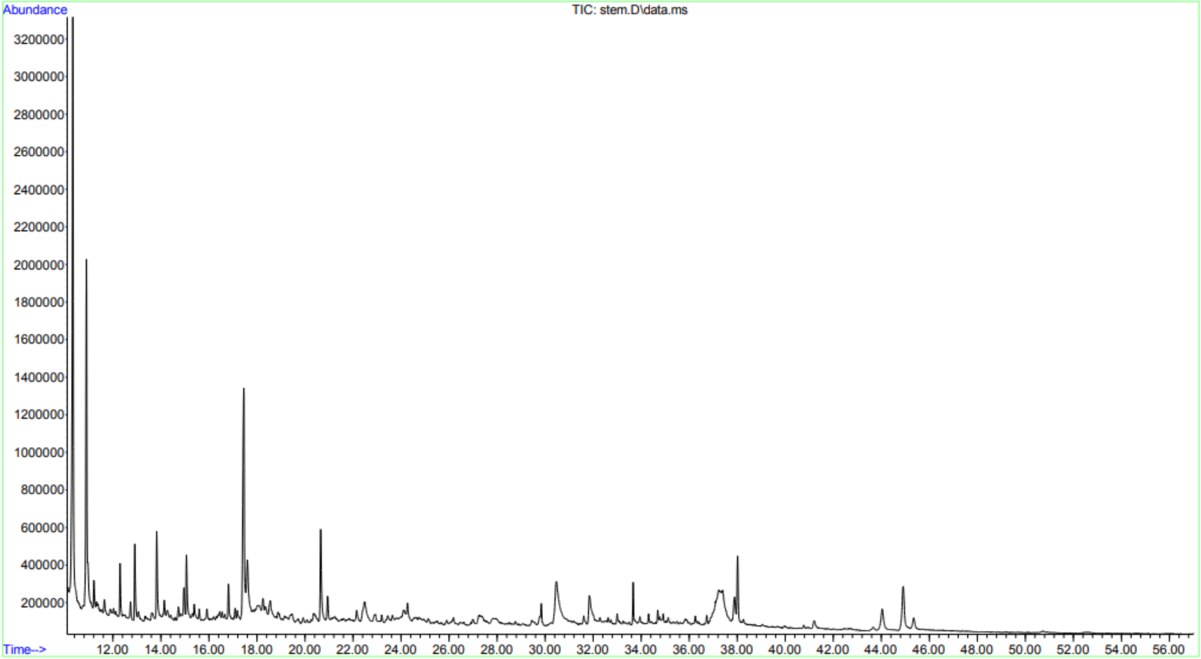

Figure S4. Chromatogram of flowering plants extract (shoots of *R. semenovii*)

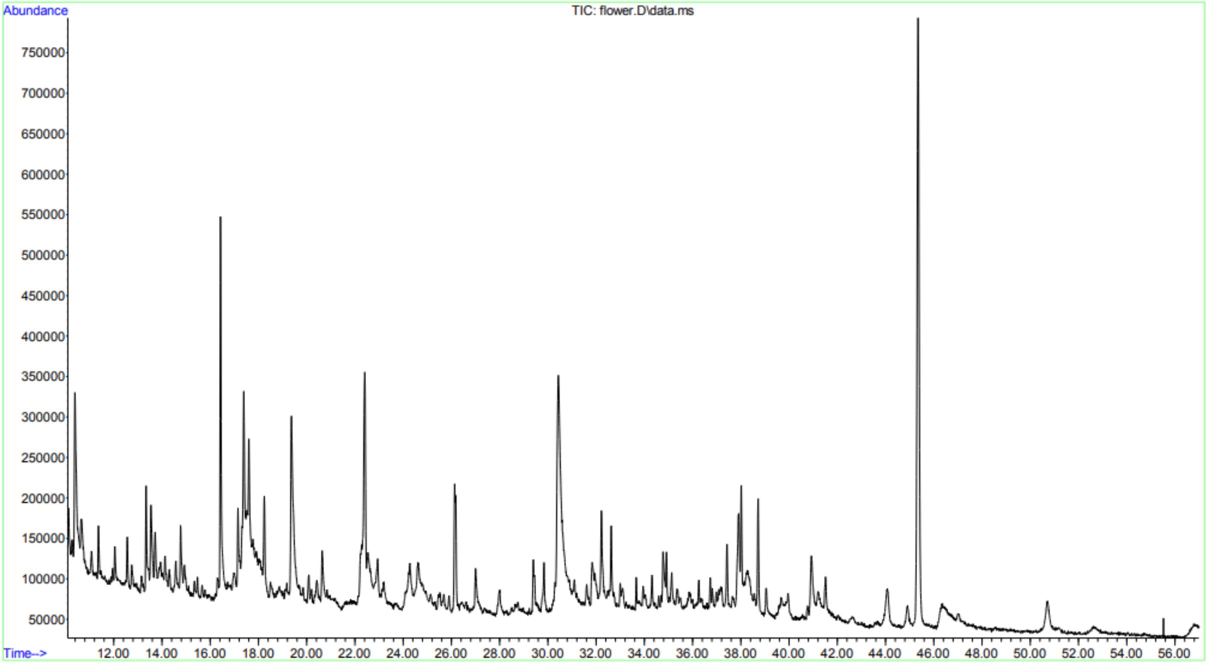

Figure S5. Chromatogram of flowering plant extract (flowers of *R. semenovii*)

Table S3. Content of SM in ripening of seeds of *R. semenovii*

	in root			in shoot		
	Content, %	Retention time, min	Iden-tifica-tion proba-bility, %	Content, %	Retention time, min	Iden-tifica-tion proba-bility, %
Fatty acids						
Oleic Acid	0.9	35.0	72	-	-	-
sum	0.9					
Fatty acids esters						
9,12-Octadecadienoic acid, ethyl ester	-	-	-	32.3	35.9	86
9,12-Octadecadienoic acid, methyl ester	4.9	34.1	85	-	-	-
9,12,15-Octadecatrienoic acid, methyl ester	0.8	34.5	75	-	-	-
Hexadecanoic acid, ethyl ester (palmitic acid ester)	-	-	-	20.5	32.2	88
Ethyl 9,12,15-octadecatrienoate	-	-	-	39.7	36.3	91
Ethyl Oleate	-	-	-	2.8	35.7	61
sum	5.7			95.3		
Monocarboxylic acid esters						
Propanoic acid, 2-oxo-, methyl ester	1.5	8.4	62	-	-	-
2-Propenoic acid, 2-methyl-, hexyl ester	0.7	23.7	64	-	-	-
sum	2.2					
Oximes						
Oxime-, methoxy-phenyl	12.3	9.1	78	-	-	-
sum	12.3					
			Ketones			
2-Propanone, 1-acetyloxy-(2)	1.5	10.4	82			
sum	1.5					
Cyclic ketones						
1,2-Cyclohexanedione	0.7	13.4	65	-	-	-
1,2-Cyclopentanedione, 3-methyl-	1.2	14.2	89	-	-	-
2-Cyclopentene-1,4-dione	2.6	10.8	68	-	-	-
2-Cyclopenten-1-one, 2-hydroxy-	2.7	11.7	92	-	-	-
sum	7.2					
Alcohols						
Cyclopropyl carbinol	2.3	17.1	78	-	-	-
Triethylene glycol	1.6	19.0	89	-	-	-
Ethanol, 2,2'-[oxybis(2,1-ethanediyloxy)]bis-	2.1	25.0	86	-	-	-
sum	6.0					

continuation of **Table S3.**						
Dioxolane derivatives						
2-t-Butyl-4-methyl-5-oxo-[1,3]dioxolane-4-carboxylic acid	2.4	21.8	66	-	-	-
sum	2.4					
Carbohydrates and its derivatives						
α-D-Glucopyranose, 4-O-β-D-galactopyranosyl-	0.9	36.1	63	-	-	-
Sucrose	2.1	24.5	70	-	-	-
sum	3.0					
Lactones						
Butyrolactone	0.7	12.7	82	-	-	-
2-Hydroxy-gamma-butyrolactone	8.6	14.7	68	-	-	-
sum	9.3					
Furan derivatives						
2,5-Furandione, 3-methyl- (9)	0.5	14.4	65	-	-	-
2(3H)-Furanone, 5-acetyldihydro-	0.6	18.1	67	-	-	-
2-Furanmethanol	0.8	9.3	79	-	-	-
Benzofuran, 2,3-dihydro-	1.0	18.4	82	-	-	-
2-Furancarboxaldehyde, 5-(hydroxymethyl)-	1.0	19.8	74	-	-	-
1,2-Ethanediol, 1-(2-furanyl)-	1.8	19.1	68	-	-	-
sum	5.2					
Pyran derivatives						
4H-Pyran-4-one, 2,3-dihydro-3,5-dihydroxy-6-methyl-	0.5	16.9	65	-	-	-
4H-Pyran-4-one, 2-ethyl-6-methyl-	0.9	15.3	63	-	-	-
sum	1.4					
Amino acids esters						
l-Alanine, N-(2-furoyl)-, ethyl ester	0.9	15.4	75	-	-	-
l-Alanine, N-(2-furoyl)-, propyl ester	0.8	20.1	69	-	-	-
sum	1.7					
Phenols						
1,2-Benzenediol, 3-methyl-	0.4	19.9	60	-	-	-
2-Methoxy-4-vinylphenol	0.6	20.4	72	-	-	-
Phenol	0.8	12.5	75	-	-	-
1,2-Benzenediol	1.2	18.0	83	-	-	-
sum	3.0					
Benzoic acid esters						
Benzoic acid, heptyl ester	0.6	29.9	63	-	-	-
sum	0.6					

continuation of **Table S3.**						
Borinic acid esters						
Borinic acid, diethyl-, methyl ester	0.9	11.8	61	-	-	-
	0.9					
Diterpenes						
3,7,11,15-Tetramethyl-2-hexadecen-1-ol (phytol)	-	-	-	4.7	34.5	60
sum				4.7		

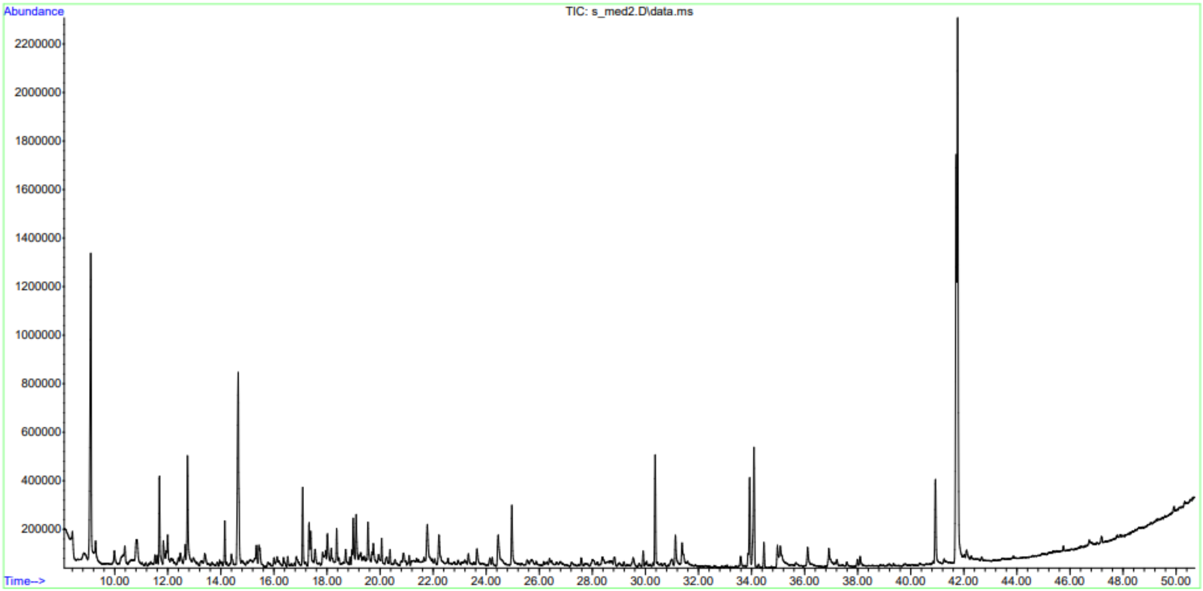

Figure S6. Chromatogram of adult plants extract (roots of *R. semenovii*)

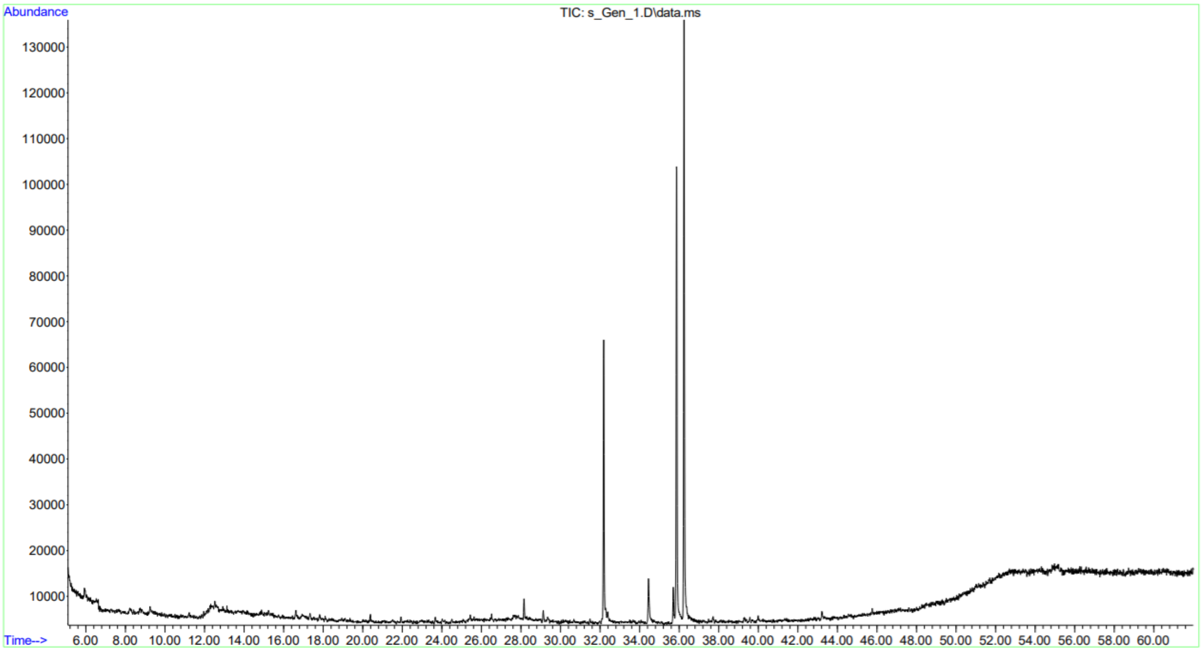

Figure S7. Chromatogram of adult plants extract (shoots of *R. semenovii*)

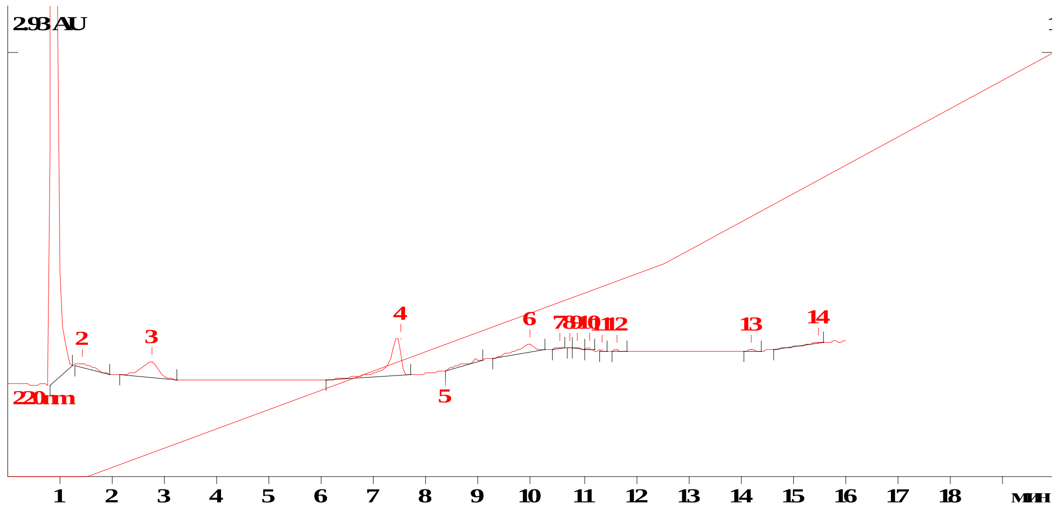

Report date: 06.12.2021 17:04:11
SAMPLE: Tyrosol 3; 3.3mg/200ml MeOH; 20mkl
Volume: 20.0 µl
COLUMN: ProntoSil-120-5-C18 AQ
Size: 2.0 x 75 mm
Part.size: 5.0 µm

Comments Tyrosol 2; in MeOH; MeOH-H3PO4 1:9-11:9-1:0 300:2500:4000
Flow: 200.00 mkl/min
Temperature: 35.0°C
Pressure: 5.3 MPa

№	Retention, mkl	Height, AU	Area, AU*mkl	Area, %	
1	177.59	16.98	316.148	91.20	
2	288.94	0.02	1.880	0.54	
3	551.76	0.10	7.910	2.28	
4	1491.06	0.27	14.014	4.04	
5	1670.57	0.00	0.857	0.25	
6	1988.64	0.06	4.172	1.20	
7	2105.90	0.00	0.135	0.04	Tyrosol
8	2138.96	0.00	0.045	0.01	
9	2169.48	0.01	0.182	0.05	
10	2217.94	0.01	0.153	0.04	
11	2263.86	0.00	0.059	0.02	
12	2326.70	0.01	0.200	0.06	
13	2837.30	0.01	0.219	0.06	
14	3084.76	0.01	0.687	0.20	
	3205.84	17.49	346.662	100.00	

Figure S8. Chromatogram of tyrosol calibration solution-3 (220nm)

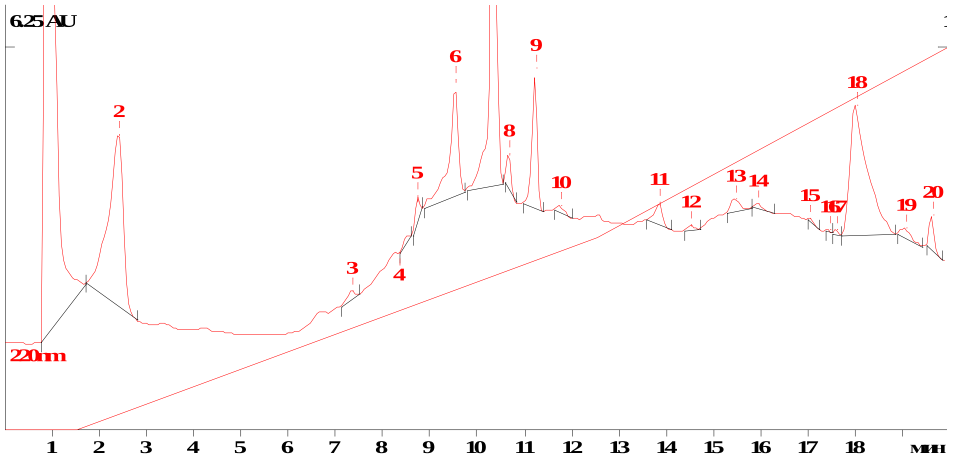

Report date: 06.12.2021 17:17:50
SAMPLE: Extr 1(upar); 155.8mg/3ml MeOH-H2O(2:1); 20mkl
Volume: 20.0 µl
COLUMN: ProntoSil-120-5-C18 AQ
Size: 2.0 x 75 mm
Part.size: 5.0 µm

Comments Extr 1(upar); in MeOH-H2O; MeOH-H3PO4 1:9-11:9-1:0 300:2500:4000
Flow: 200.00 mkl/min
Temperature: 35.0°C
Pressure: 5.3 MPa

№	Retention, mkl	Height, AU	Area, AU*mkl	Area, %	
1	177.73	19.11	826.213	50.10	
2	483.23	2.83	207.528	12.59	
3	1466.25	0.14	4.807	0.29	
4	1670.68	-0.02	0.916	0.06	
5	1748.03	0.37	6.392	0.39	
6	1908.02	1.92	81.375	4.93	
7	2069.15	7.30	211.454	12.82	Tyrosol
8	2132.62	0.62	12.544	0.76	
9	2242.82	2.30	55.963	3.39	
10	2348.00	0.11	3.823	0.23	
11	2768.70	0.37	13.470	0.82	
12	2902.81	0.08	2.636	0.16	
13	3086.23	0.01	8.630	0.52	
14	3185.77	0.09	2.935	0.18	
15	3403.81	0.06	1.314	0.08	
16	3487.08	0.05	1.004	0.06	
17	3513.10	0.08	1.137	0.07	
18	3599.01	2.13	182.897	11.09	
19	3808.66	0.17	8.568	0.52	
20	3922.68	0.61	15.401	0.93	
	3981.52	38.57	1649.007	100.00	

Dry residue content in extract 1: (100* $m_{\text{dry residue}}$) / m_{extract1} = (100* 0.1558)/ 44.854 = 0.347 %
Tyrosol content in dry part of extract 1: 100* $_{\text{tyrosol on chrom. of extr. 1}}$ * $C_{\text{tyrosol in calibr. sol 3}}$) / $_{\text{tyrosol on chrom. of calibr. sol. 3}}$ * $C_{\text{tyrosol in calibr. sol. 3}}$ = ((100* 4.807* 3.3/200)/(14.014* 155.8/3) = 0.0109% (calibr. sol. 3 - 220 nm)
Salidroside content in the dry part of extract 2: $C_{\text{tyrosol in the dry part of extr. 1}}$ 2.17 =0.024%

Figure S9. Determination of salidroside content in the shoot of an immature plants of *R. semenovii*

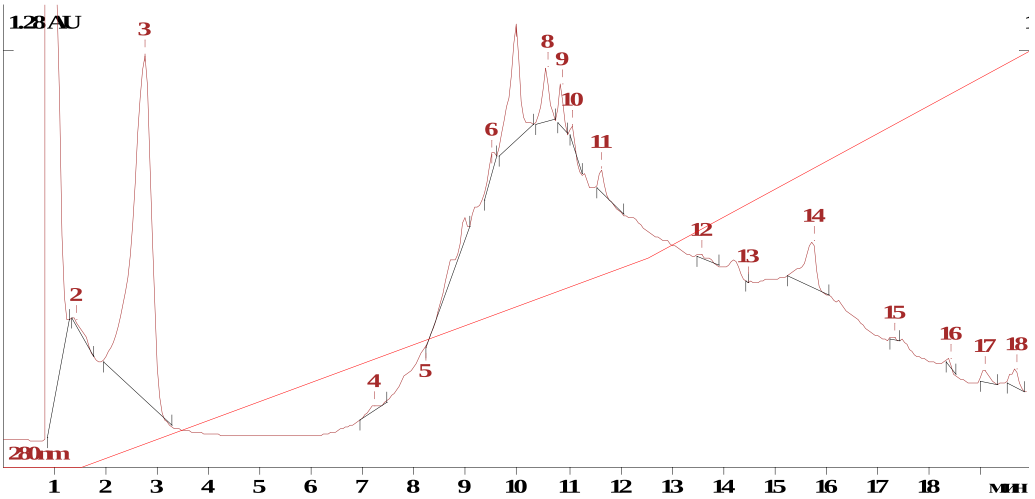

Report date: 07.12.2021 12:17:49
SAMPLE: Extr 2(upar); 215mg/20ml MeOH-H2O; 20mkl
Volume: 20.0 µl
COLUMN: ProntoSil-120-5-C18 AQ
Size: 2.0 x 75 mm
Part.size: 5.0 µm

Comments Extr 2(upar); in MeOH-H2O; MeOH-H3PO4 1:9-11:9-1:0 300:2500:4000
Flow: 200.00 mkl/min
Temperature: 35.0°C
Pressure: 5.4 MPa

№	Retention, mkl	Height, AU	Area, AU*mkl	Area, %	
1	185.47	18.71	553.611	80.03	
2	285.29	0.02	1.342	0.19	
3	553.58	1.07	92.371	13.35	
4	1437.99	0.02	0.546	0.08	
5	1644.51	0.00	2.036	0.29	
6	1900.71	0.04	0.875	0.13	
7	1995.12	0.36	17.289	2.50	Tyrosol
8	2111.58	0.17	5.176	0.75	
9	2167.61	0.14	2.617	0.38	
10	2215.54	0.08	1.674	0.24	
11	2325.51	0.08	2.041	0.30	
12	2711.65	0.01	0.566	0.08	
13	2888.30	0.00	-0.003	0.00	
14	3145.98	0.15	7.867	1.14	
15	3455.56	0.01	0.165	0.02	
16	3673.43	0.03	0.453	0.07	
17	3812.42	0.04	1.306	0.19	
18	3933.39	0.06	1.796	0.26	
	3981.18	20.99	691.734	100.00	

Dry residue content in extract 2: (100* 0.2146)/ 54.7186 = 0.392 %
Tyrosol content in dry part of extract 2: (100* 0.546* 3.3/200)/(2.818* 215/20) = 0.0297% (calibr. sol. 3 – 280 nm)
Salidroside content in the dry part of extract 2:0.065%

Figure S10. Determination of salidroside content in the root of an immature plants of *R.semenovii*

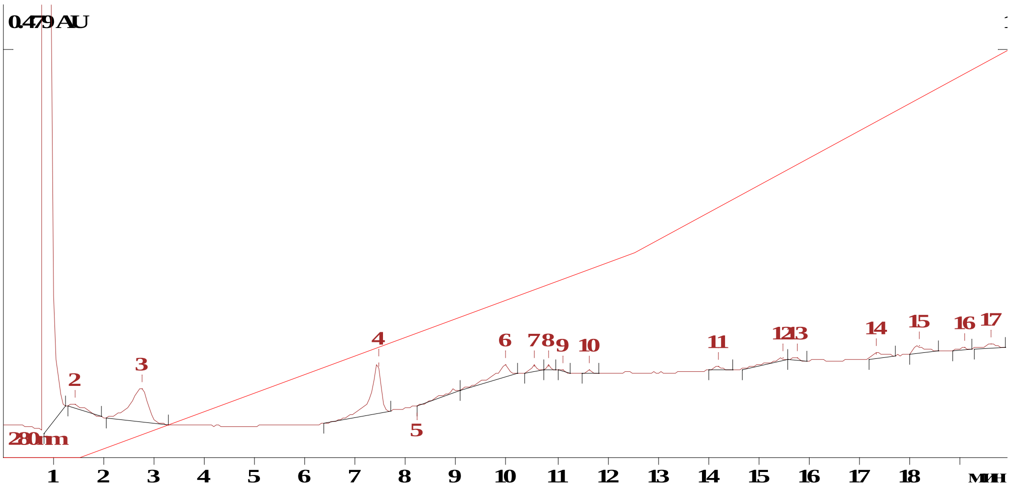

Report date: 07.12.2021 12:22:23
SAMPLE: Tyrosol 2; 3.3mg/200ml MeOH; 20mkl
Volume: 20.0 µl
COLUMN: ProntoSil-120-5-C18 AQ
Size: 2.0 x 75 mm
Part.size: 5.0 µm

Comments Tyrosol 2; in MeOH; MeOH-H3PO4 1:9-11:9-1:0 300:2500:4000
Flow: 200.00 mkl/min
Temperature: 35.0°C
Pressure: 5.3 MPa

№	Retention, mkl	Height, AU	Area, AU*mkl	Area, %	
1	179.71	3.80	82.861	89.93	
2	287.20	0.00	0.370	0.40	
3	551.59	0.04	3.114	3.38	
4	1487.92	0.06	2.818	3.06	
5	1644.55	0.00	0.106	0.12	
6	1989.65	0.01	0.879	0.95	
7	2110.01	0.01	0.187	0.20	Tyrosol
8	2167.30	0.01	0.143	0.16	
9	2215.61	0.00	0.065	0.07	
10	2326.14	0.00	0.103	0.11	
11	2836.73	0.01	0.170	0.18	
12	3083.97	0.00	0.132	0.14	
13	3147.42	0.00	0.116	0.13	
14	3462.62	0.01	0.331	0.36	
15	3625.04	0.01	0.442	0.48	
16	3811.67	0.00	0.052	0.06	
17	3919.63	0.00	0.251	0.27	
	3981.28	3.97	92.139	100.00	

Figure S11. Chromatogram of tyrosol calibration solution (280nm)

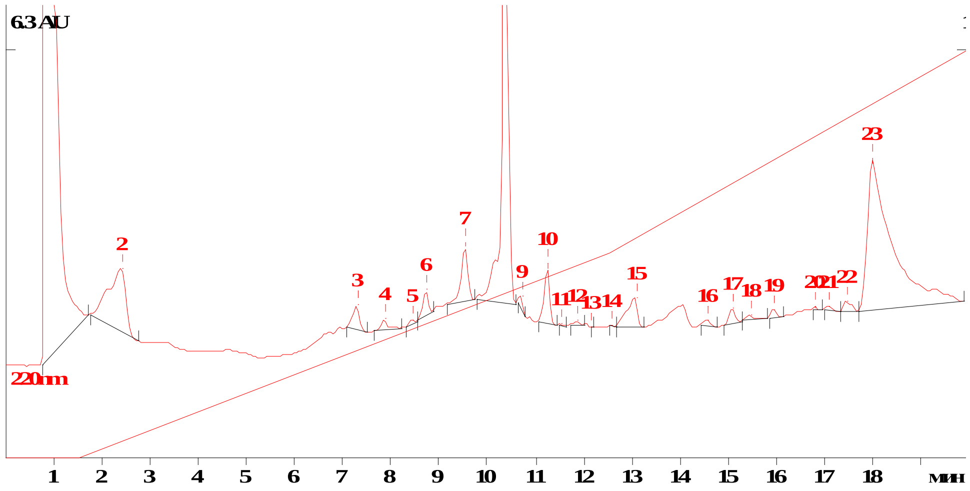

Report date: 06.12.2021 17:16:17
SAMPLE: Extr 3(upar); 138mg/3ml MeOH-H2O(2:1); 20mkl
Volume: 20.0 µl
COLUMN: ProntoSil-120-5-C18 AQ
Size: 2.0 x 75 mm
Part.size: 5.0 µm
Comments Extr 3(upar); in MeOH-H2O; MeOH-H3PO4 1:9-11:9-1:0 300:2500:4000
Flow: 200.00 mkl/min
Temperature: 35.0°C
Pressure: 5.3 MPa

№	Retention, mkl	Height, AU	Area, AU*mkl	Area, %	
1	176.23	18.28	809.209	54.77	
2	480.64	0.99	83.059	5.62	
3	1456.73	0.38	12.771	0.86	
4	1572.13	0.17	5.136	0.35	
5	1688.47	0.06	1.092	0.07	
6	1745.92	0.41	9.593	0.65	
7	1908.68	0.91	27.405	1.85	Tyrosol
8	2075.96	6.02	181.736	12.30	
9	2138.76	0.21	3.357	0.23	
10	2247.35	0.95	23.498	1.59	
11	2300.41	0.01	0.202	0.01	
12	2371.96	0.07	2.020	0.14	
13	2430.52	0.00	-0.013	0.00	
14	2513.80	0.02	0.197	0.01	
15	2610.87	0.47	21.679	1.47	
16	2909.15	0.10	3.489	0.24	
17	3016.30	0.23	6.176	0.42	
18	3084.14	0.07	2.135	0.14	
19	3187.87	0.13	2.913	0.20	
20	3356.93	0.06	0.926	0.06	
21	3410.88	0.07	2.262	0.15	
22	3488.76	0.16	6.072	0.41	
23	3597.83	2.34	272.647	18.45	
	3981.48	32.10	1477.587	100.00	

Dry residue content in extract 3: (100* 0.1376)/ 49.9366 = 0.276 %
Tyrosol content in dry part of extract 3: (100* 12.771* 3.3/200)/(14.014* 138/3) = 0.0327% (calibr. sol. 3 - 220 nm)
Salidroside content in the dry part of extract 3:0.071%

Figure S12. Determination of salidroside content during the flowering in the shoot of the plants of *R. semenovii*

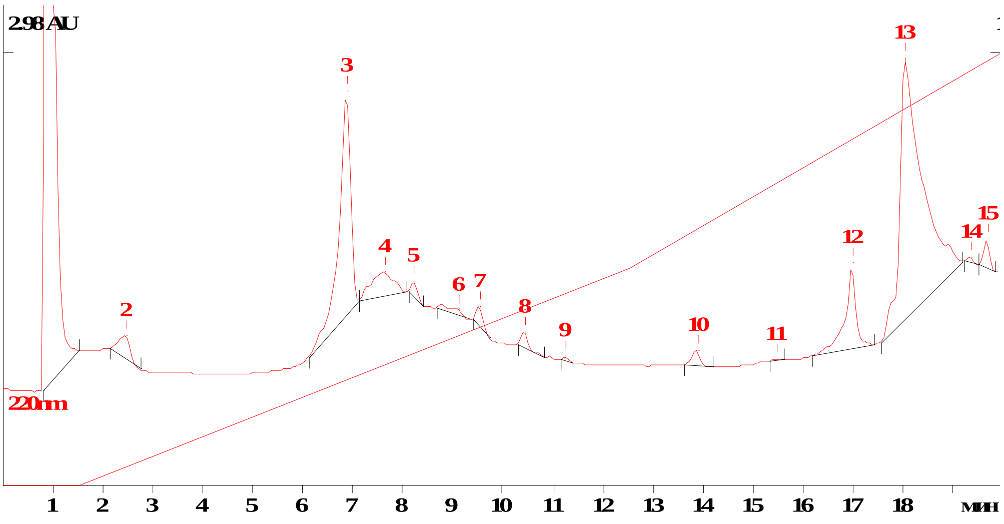

Report date: 06.12.2021 17:10:49
SAMPLE: Tyrosol 2; 3.3mg/20ml MeOH; 20mkl
Volume: 20.0 µl
COLUMN: ProntoSil-120-5-C18 AQ
Size: 2.0 x 75 mm
Part.size: 5.0 µm

Comments Tyrosol 2; in MeOH; MeOH-H3PO4 1:9-11:9-1:0 300:2500:4000
Flow: 200.00 mkl/min
Temperature: 35.0°C
Pressure: 5.0 MPa

№	Retention, mkl	Height, AU	Area, AU*mkl	Area, %	
1	180.24	18.30	586.421	63.16	
2	492.53	0.16	8.668	0.93	
3	1375.70	1.55	80.025	8.62	
4	1524.65	0.17	17.219	1.85	
5	1645.42	0.12	3.189	0.34	
6	1818.39	0.04	3.341	0.36	
7	1908.40	0.16	4.843	0.52	Tyrosol
8	2085.03	0.11	3.453	0.37	
9	2248.24	0.03	0.663	0.07	
10	2772.97	0.10	3.575	0.39	
11	3087.52	0.01	0.362	0.04	
12	3395.23	0.59	28.326	3.05	
13	3606.74	1.79	182.070	19.61	
14	3867.17	0.05	1.332	0.14	
15	3932.19	0.20	5.016	0.54	
	3981.63	23.37	928.504	100.00	

Figure S13. Chromatogram of tyrosol calibration solution-2 (220nm)

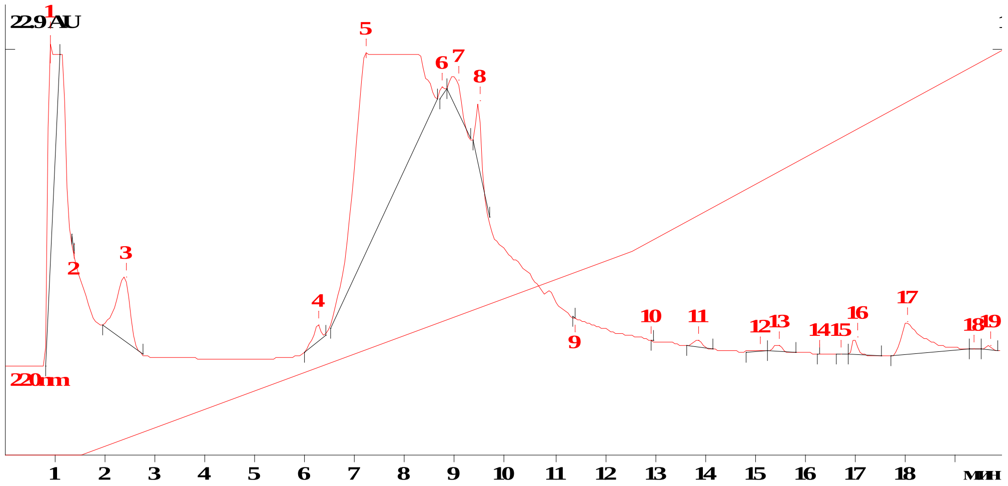

Report date: 06.12.2021 17:09:06
SAMPLE: Extr 4(upar); 169.8mg/3ml MeOH-H_2O(2:1); 20mkl
Volume: 20.0 µl
COLUMN: ProntoSil-120-5-C18 AQ
Size: 2.0 x 75 mm
Part.size: 5.0 µm
Comments Extr 4(upar); in MeOH-H2O; MeOH-H3PO4 1:9-11:9-1:0 300:2500:4000
Flow: 200.00 mkl/min
Temperature: 35.0°C
Pressure: 5.0 MPa

№	Retention, mkl	Height, AU	Area, AU*mkl	Area, %	
1	179.39	13.04	340.195	9.88	
2	270.65	-0.06	-0.329	-0.01	
3	479.76	3.59	232.402	6.75	
4	1250.88	0.86	27.442	0.80	Tyrosol
5	1445.62	11.01	2381.572	69.20	
6	1743.63	0.43	6.033	0.18	
7	1807.77	1.76	85.933	2.50	
8	1896.01	4.02	109.105	3.17	
9	2274.53	0.00	-0.012	0.00	
10	2581.62	0.00	-0.016	0.00	
11	2769.09	0.37	14.597	0.42	
12	3011.84	0.03	1.311	0.04	
13	3091.84	0.40	16.502	0.48	
14	3247.84	0.00	-0.006	0.00	
15	3335.29	0.01	0.292	0.01	
16	3396.28	1.01	25.233	0.73	
17	3604.27	1.85	192.118	5.58	
18	3869.58	0.05	1.013	0.03	
19	3931.22	0.28	7.663	0.22	
	3981.73	38.77	3441.775	100.00	

Dry residue content in extract 4: (100* 0.4367)/ 42.8450 = 1.02 %
Tyrosol content in dry part of extract 4: (100* 27.442* 3.3/20)/(80.025* 169.8/3) = 0.0999% (calibr. sol. 2 – 220nm)
Salidroside content in the dry part of extract 4:0.217%

Figure S14. Determination of salidroside content during the flowering in the root of the plants of *R. semenovii*

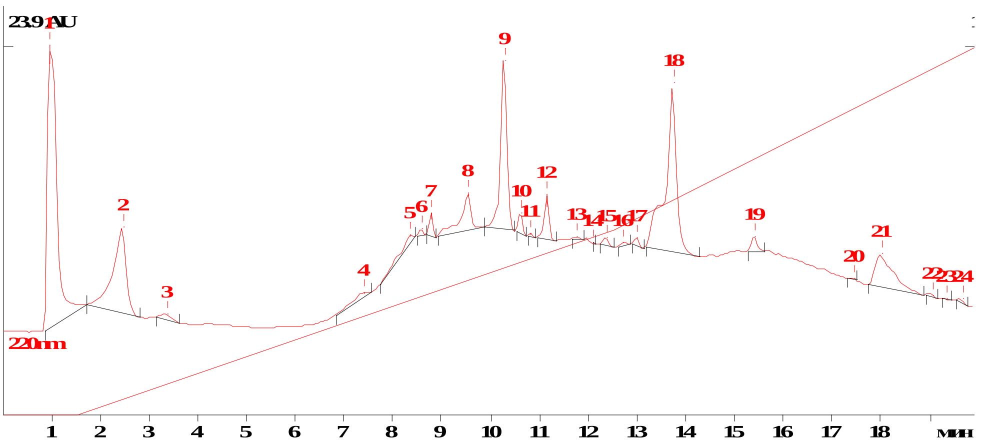

Report date: 06.12.2021 17:13:34
SAMPLE: Extr 5(upar); 229mg/3ml MeOH-H2O(2:1); 20mkl
Volume: 20.0 µl
COLUMN: ProntoSil-120-5-C18 AQ
Size: 2.0 x 75 mm
Part.size: 5.0 µm
Comments Extr 5(upar); in MeOH-H2O; MeOH-H3PO4 1:9-11:9-1:0 300:2500:4000
Flow: 200.00 mkl/min
Temperature: 35.0°C
Pressure: 7.8 MPa

№	Retention, mkl	Height, AU	Area, AU*mkl	Area, %	
1	192.92	18.35	811.120	30.59	
2	487.58	5.59	358.530	13.52	
3	673.22	0.35	16.845	0.64	
4	1476.93	0.29	21.291	0.80	
5	1669.20	0.47	29.496	1.11	
6	1714.81	0.46	8.356	0.32	
7	1756.73	1.56	28.352	1.07	Tyrosol
8	1905.35	2.43	121.169	4.57	
9	2053.97	11.14	315.598	11.90	
10	2122.27	1.44	30.918	1.17	
11	2161.47	0.24	3.570	0.13	
12	2230.93	2.96	72.243	2.72	
13	2351.50	0.14	4.432	0.17	
14	2416.68	-0.01	-0.045	0.00	
15	2472.90	0.52	11.136	0.42	
16	2540.25	0.20	5.253	0.20	
17	2599.77	0.57	12.563	0.47	
18	2744.40	11.07	576.668	21.75	
19	3081.01	1.02	28.182	1.06	
20	3489.60	0.07	1.275	0.05	
21	3598.08	2.15	181.545	6.85	
22	3807.61	0.14	3.816	0.14	
23	3863.03	0.08	1.475	0.06	
24	3928.11	0.26	7.958	0.30	
	3979.18	61.51	2651.834	100.00	

Dry residue content in extract 5: (100* 0.2294)/ 48.1120 = 0.477 %
Tyrosol content in the dry part of extract 5: (100* 21.991* 3.3/20)/(80.025* 229/3) = 0.0575% (calibr. sol. 2 – 220 nm)
Salidroside content in the dry part of extract 5: 0.125%

Figure S15. Determination of salidroside content during ripening of seeds in the shoot of the plants of *R. semenovii*

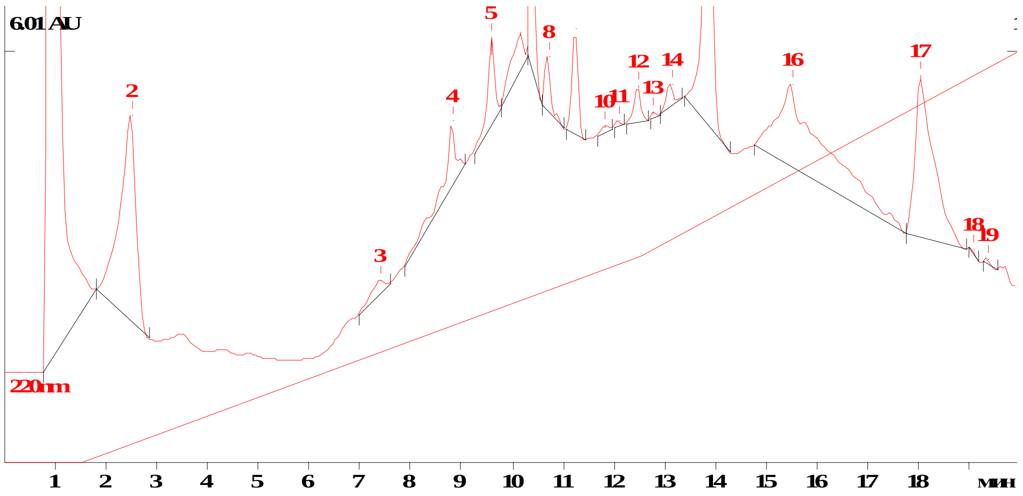

Report date: 06.12.2021 17:07:21
SAMPLE: Extr 6-2(upar); 245mg/5ml MeOH-H2O(2:1); 20mkl
Volume: 20.0 µl
COLUMN: ProntoSil-120-5-C18 AQ
Size: 2.0 x 75 mm
Part.size: 5.0 µm
Comments Extr 6-2(upar); in MeOH-H2O; MeOH-H3PO4 1:9-11:9-1:0 300:2500:4000
Flow: 200.00 mkl/min
Temperature: 35.0°C
Pressure: 5.5 MPa

№	Retention, mkl	Height, AU	Area, AU*mkl	Area, %	
1	178.00	19.13	952.994	43.07	
2	498.45	3.03	224.982	10.17	
3	1475.65	0.21	11.331	0.51	
4	1761.65	0.97	46.873	2.12	
5	1919.94	1.25	37.794	1.71	
6	2030.23	0.53	29.772	1.35	
7	2080.57	3.17	69.888	3.16	Tyrosol
8	2139.65	0.85	21.963	0.99	
9	2246.87	1.57	40.439	1.83	
10	2361.50	0.09	2.970	0.13	
11	2413.05	0.08	1.595	0.07	
12	2494.36	0.54	16.841	0.76	
13	2553.78	0.08	1.454	0.07	
14	2618.50	0.33	9.475	0.43	
15	2766.16	7.48	267.651	12.10	
16	3093.43	1.20	272.938	12.34	
17	3604.68	2.36	199.703	9.03	
18	3811.66	0.08	2.049	0.09	
19	3865.93	0.08	1.700	0.08	
	3980.82	43.01	2212.410	100.00	

Dry residue content in extract 6: (100* 0.2446)/ 48.5895 = 0.503 %
Tyrosol content in dry part of extract 6: (100* 11.331* 3.3/200)/(14.014* 245/5) = 0.0272% (calibr. sol. 3 – 220 nm)
Salidroside content in the dry part of extract 6: 0.059%

Figure S16. Determination of salidroside content during ripening of seeds in the root of the plants of *R. semenovii*
